# Supplementary material for: CRISPR antiphage defence mediated by the cyclic nucleotide-binding membrane protein Csx23
Source: Nucleic Acids Res. 2024 Mar 13;52(6):2761–75. doi: 10.1093/nar/gkae167 (PMC11014256; doi:10.1093/nar/gkae167)
Supplement: gkae167_Supplemental_File [file gkae167_supplemental_file.pdf]

Supporting Information

CRISPR antiphage defence mediated by the cyclic nucleotide-binding membrane protein Csx23

AUTHORS

Sabine Grüschow[1], Stuart McQuarrie[1], Katrin Ackermann[2], Stephen McMahon[1], Bela E. Bode[2], Tracey M. Gloster[1*], Malcolm F. White[1*]

1 Biomedical Sciences Research Complex, School of Biology, University of St Andrews, St Andrews, Fife KY16 9ST, UK.

2 Biomedical Sciences Research Complex, School of Chemistry, Centre of Magnetic Resonance, University of St Andrews, St Andrews, Fife, KY16 9ST, UK.

* To whom correspondence should be addressed. Tel +44-1334 463432; email: mfw2@st-andrews.ac.uk; tmg@st-andrews.ac.uk

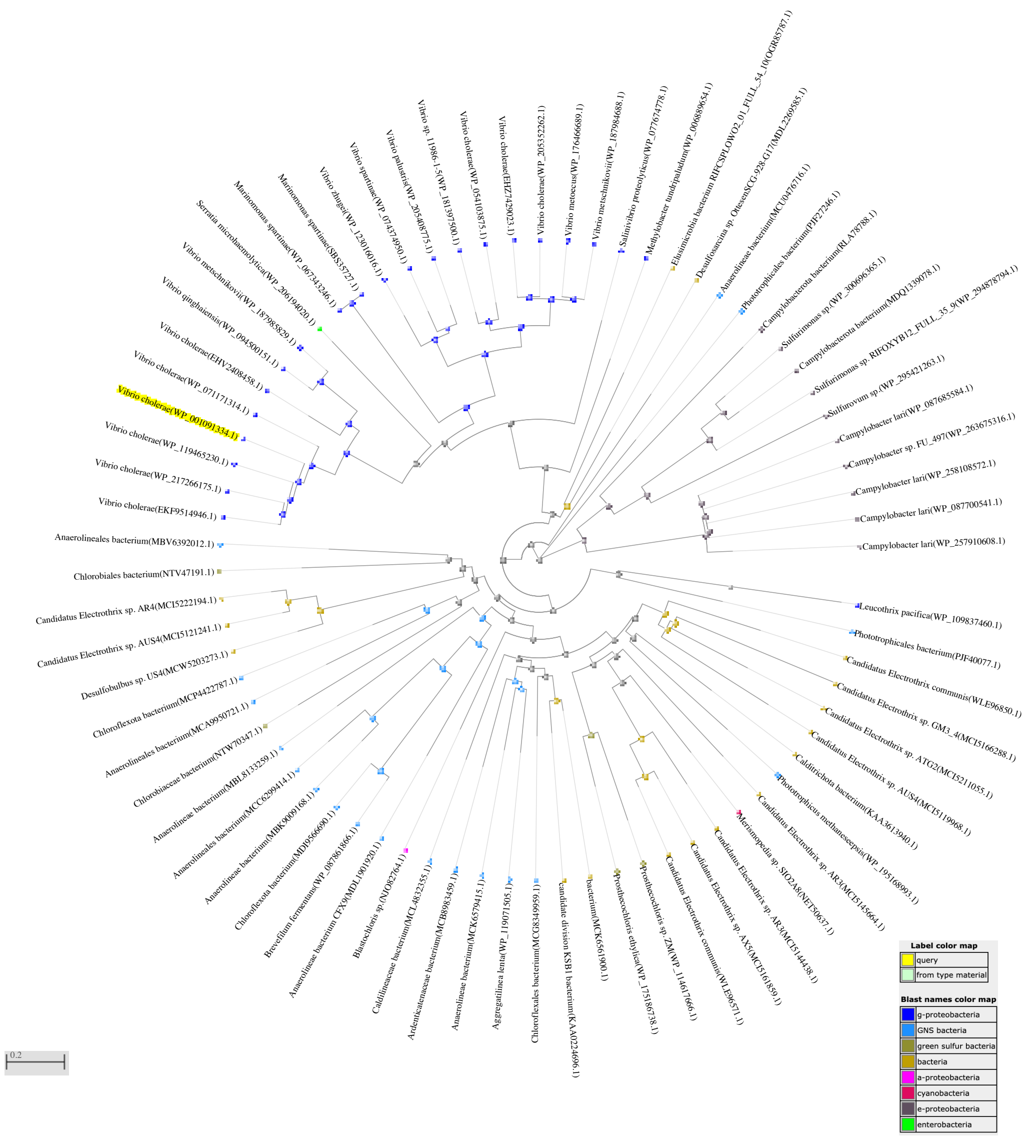

Figure S1. Distance tree of PSI-BLAST results using Csx23 (WP_001091334.1, highlighted) as query (NCBI website). The tree is based on pairwise alignments with fast minimum evolution (Grishin distance model, 0.85 maximum sequence difference) as method for tree construction.

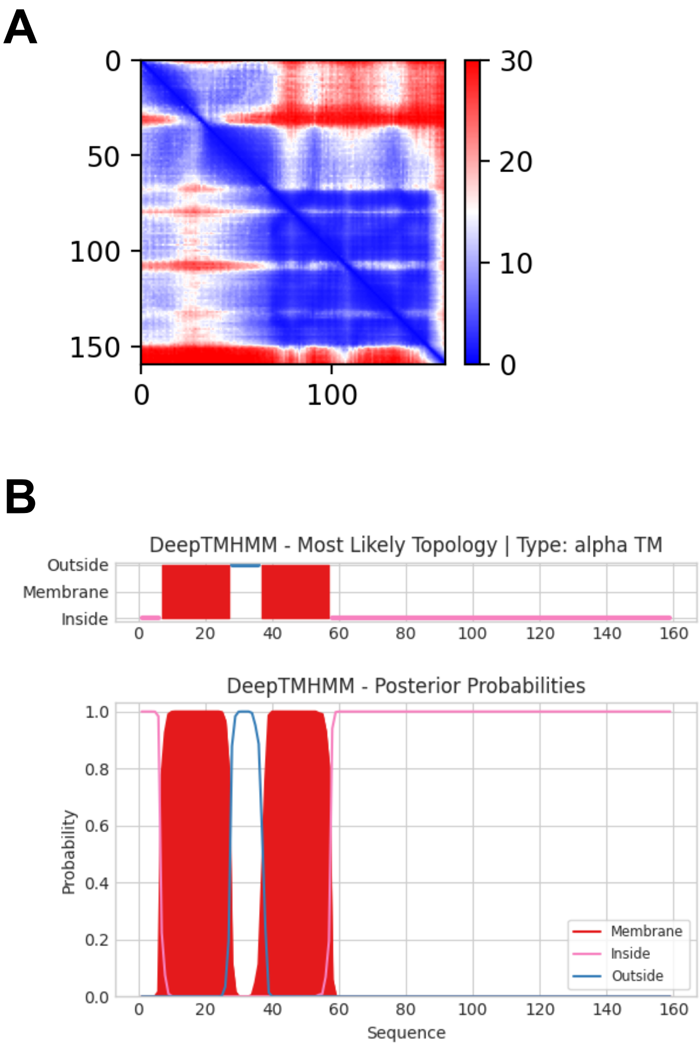

Figure S2. *In silico* analyses of Csx23. **A:** Predicted Aligned Error (PAE) plot output from AF2 for FL Csx23. The colour at (x, y) indicates the expected position error at residue x (x axis) if the predicted and true structures were aligned on residue y (y axis). Coloured from blue (low error) to red (high error) as indicated on side bar in Å. This plot shows the residue positional predictions within each domain have low error, but predictions of the residue positions between the two domains is higher. **B:** Prediction of transmembrane domains using DeepTMHMM indicating very strongly a membrane-spanning N-terminal domain and a soluble, cytosolic C-terminal domain.

CTD Csx23

Hydrodynamic Radius

Z-Average (± SD) (d.nm): 3.404 ± 1.532

Polydispersity Index: 0.203

Estimated MW (± SD) (KDa): 11.7 ± 5.3

%Polydispersity: 45.0

Sample Polydispersity: Polydisperse

Distribution Results

	Mode ± SD (nm)	%Pd	Est. MW (KDa) (Mean ± SD)*	% Intensity	% Mass	Peak Polydispersity
**Peak 1:**	3.878 ± 1.812	42.0	20.3 ± 8.5	100.0	100.0	Polydisperse
**Peak 2**	0.000 ± 0.000	0	0.0 ± 0.0	0.0	0.0	
**Peak 3:**	0.000 ± 0.000	0	0.0 ± 0.0	0.0	0.0	

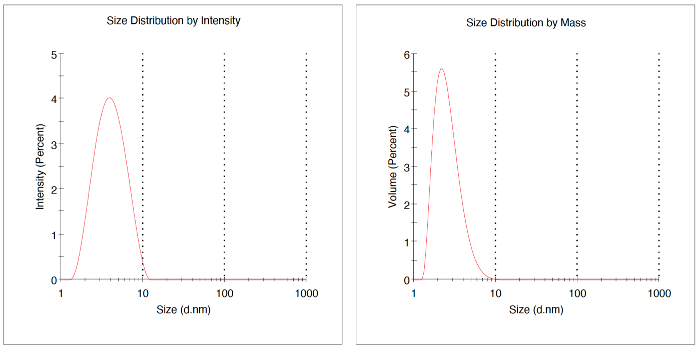

FL Csx23

Hydrodynamic Radius

Z-Average (± SD) (d.nm): 10.18 ± 2.080

Polydispersity Index: 0.042

Estimated MW (± SD) (KDa): 152 ± 31.0

%Polydispersity: 20.4

Sample Polydispersity: Monodisperse

Distribution Results

	Mode ± SD (nm)	%Pd	Est. MW (KDa) (Mean ± SD)*	% Intensity	% Mass	Peak Polydispersity
**Peak 1:**	10.23 ± 2.058	19.4	167.0 ± 32.4	100.0	100.0	Monodisperse
**Peak 2**	0.000 ± 0.000	0	0.0 ± 0.0	0.0	0.0	
**Peak 3:**	0.000 ± 0.000	0	0.0 ± 0.0	0.0	0.0	

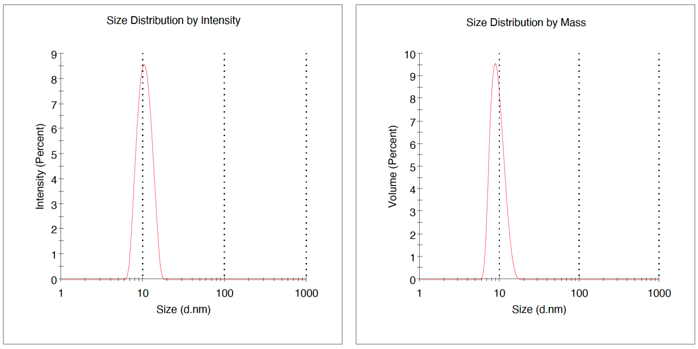

Figure S3. Dynamic light scattering (DLS) analysis of Csx23. The molecular weight (MW) was calculated from the hydrodynamic radius using the Zetasizer software assuming a globular shape. The soluble, C-terminal domain (CTD, top) of Csx23 was measured in the absence of detergent; the size measured by DLS fits well with the CTD being monomeric (expected MW 10.4 kDa). Full length (FL, bottom) Csx23 was measured in the presence of 0.1% DDM; the size measured for the full length detergent:protein complex is at least 10 times larger than that obtained for the CTD. This may suggest an oligomeric form for FL Csx23, however, the presence of detergent precludes further interpretation.

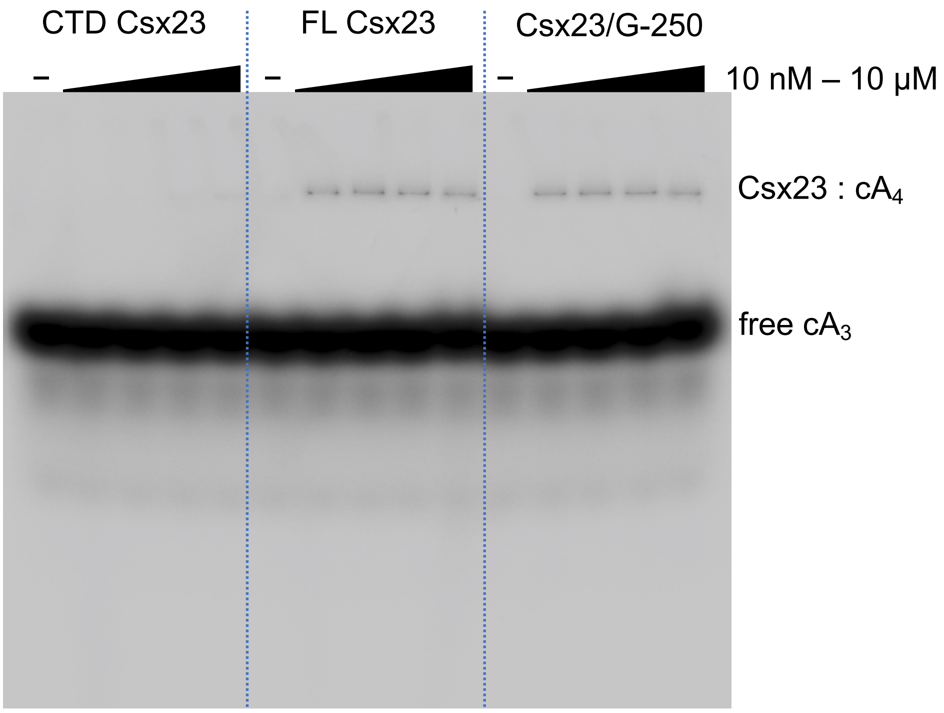

Figure S4. EMSA with radiolabelled VmeCmr-derived cA_3 and FL Csx23 or Csx23 CTD. A 10-fold serial dilution of Csx23 was incubated with approximately 1.5 µM [^{32}P]-cOA each. Csx23/G-250: Coomassie G-250 was added prior to loading.

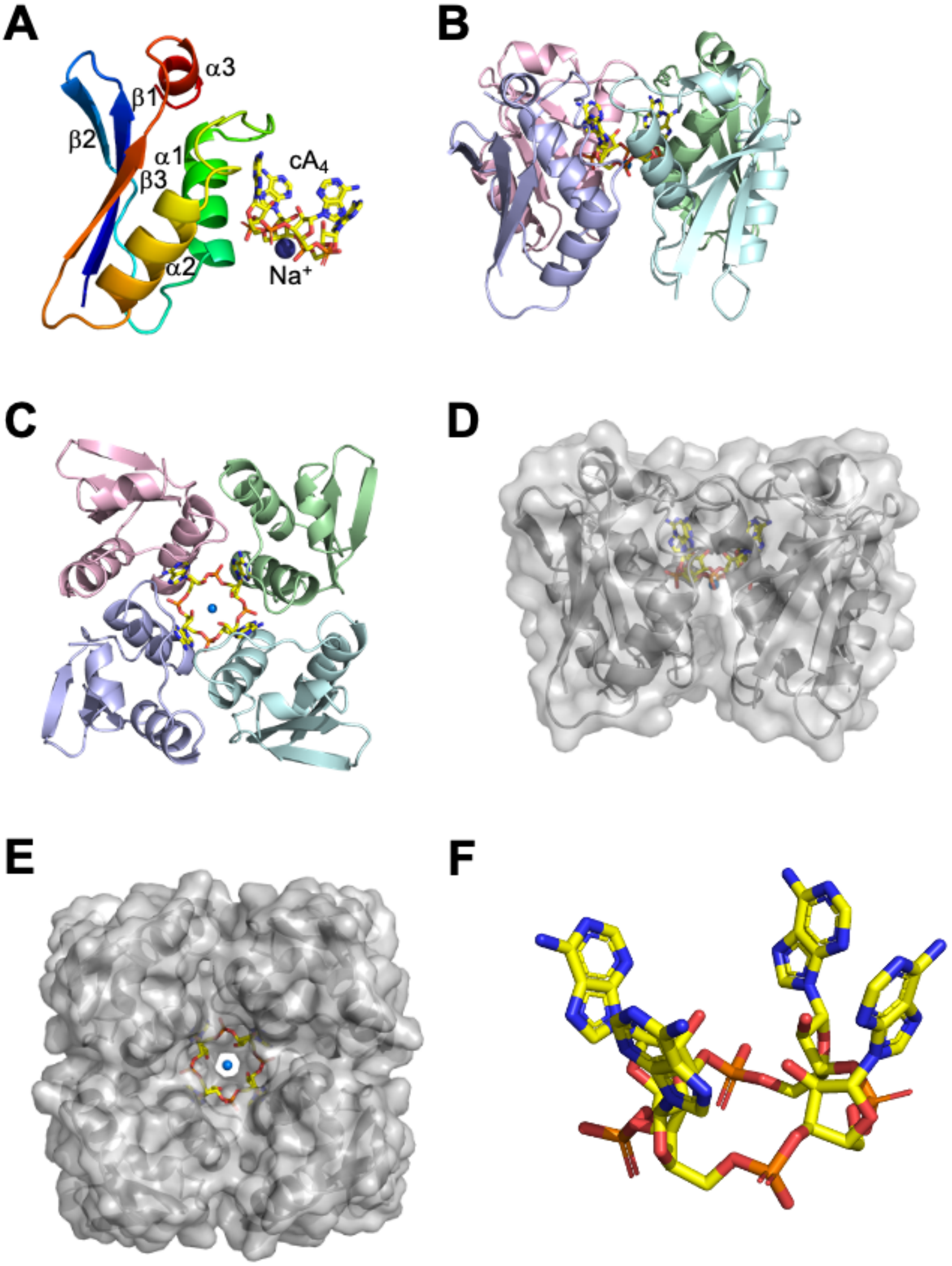

Figure S5. Structure of Csx23 CTD. **A**: Cartoon representation of a single subunit of Csx23 CTD coloured from N- (blue) to C-terminus (red), with secondary structure elements labelled (α, α-helix; β, β-strand; numbering of each element from N- to C-terminus). cA_4 is shown in sticks (coloured by element with carbon in yellow) and the sodium ion as a blue sphere for orientation. **B**: 'Side' view and **C**: 'top' view of tetrameric Csx23 CTD, shown in cartoon representation with each subunit in a different colour. cA_4 is shown in sticks (coloured by element with carbon in yellow) and the sodium ion as a blue sphere. **D**: 'Side' view and **E**: 'top' view of tetrameric Csx23 CTD shown in surface representation. Colouring as in panels **B**/**C**. **F**: Stick representation of cA_4, coloured by element with carbon in yellow.

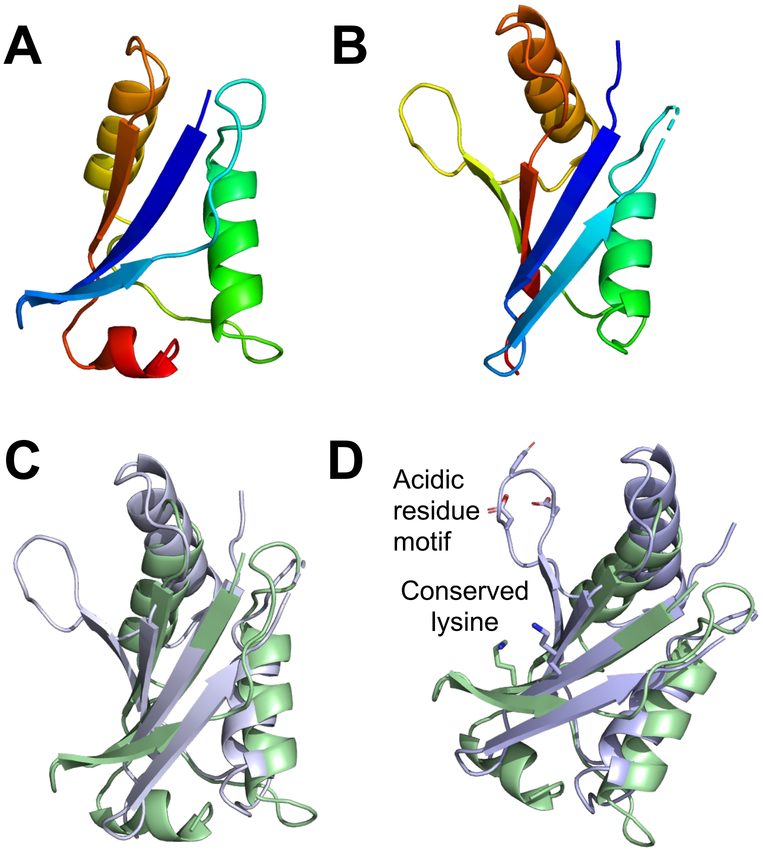

Figure S6. Comparison of the structures of Csx23 CTD and the PB1 domain from protein kinase C zeta type. **A**: Cartoon representation of a single subunit of Csx23 CTD coloured from N- (blue) to C-terminus (red). **B**: Cartoon representation of the PB1 domain from protein kinase C zeta type from rat (PDB: 4MJS) coloured from N- (blue) to C-terminus (red). **C**: Superimposition of Csx23 CTD (green cartoon) and the PB1 domain from protein kinase C zeta type (light blue cartoon). **D**: Superimposition of Csx23 CTD (green cartoon) and the PB1 domain from protein kinase C zeta type (light blue cartoon), highlighting a conserved lysine residue at the end of the first beta-strand in each protein, and the additional region containing acidic residues present only in the PB1 domain.

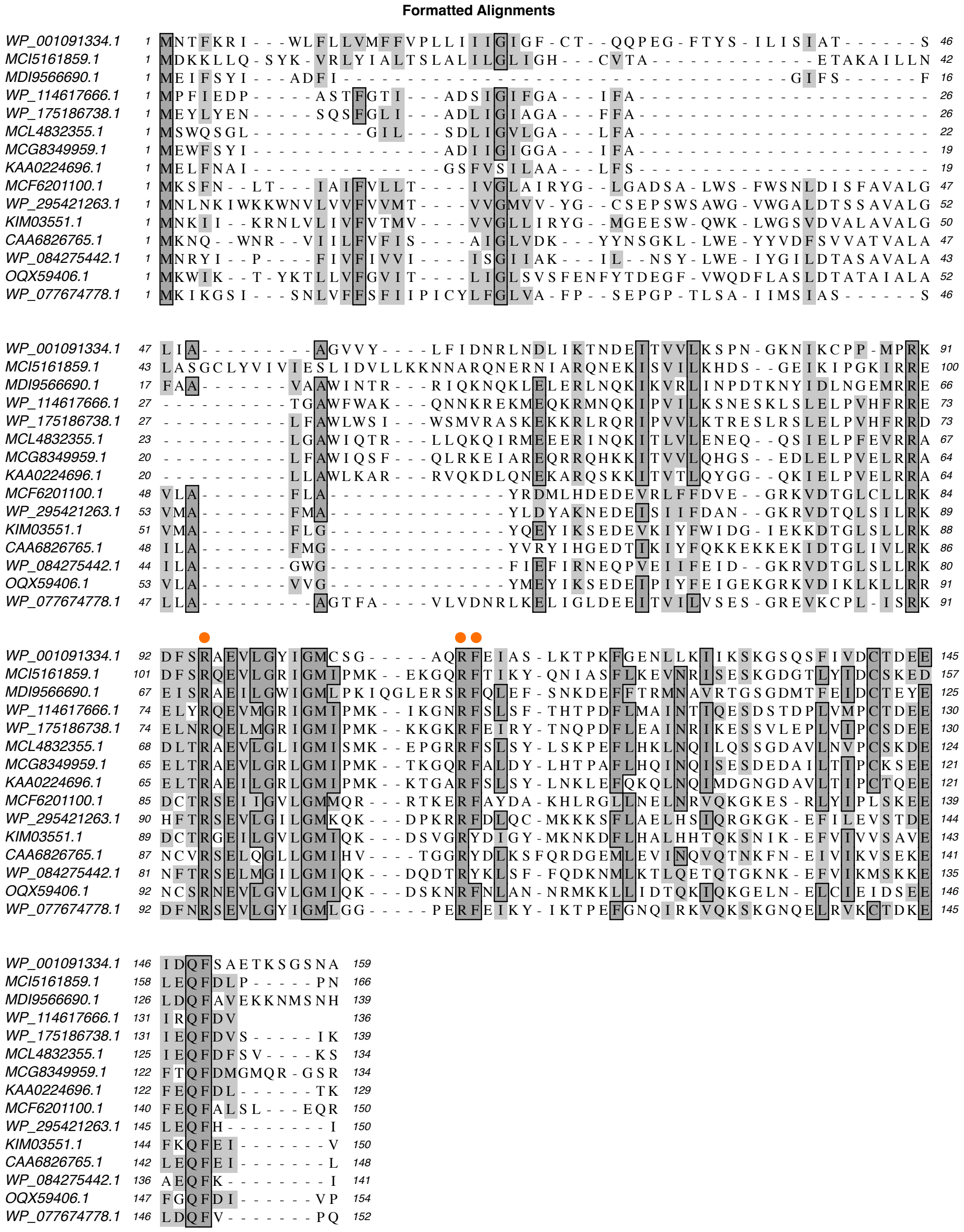

Figure S7. Multiple sequence alignment of *V. cholerae* Csx23 (WP_001091334.1) and diverse homologues, aligned using MUSCLE. Residues targeted by mutagenesis (R95, R110, and F111) are marked by a dot.

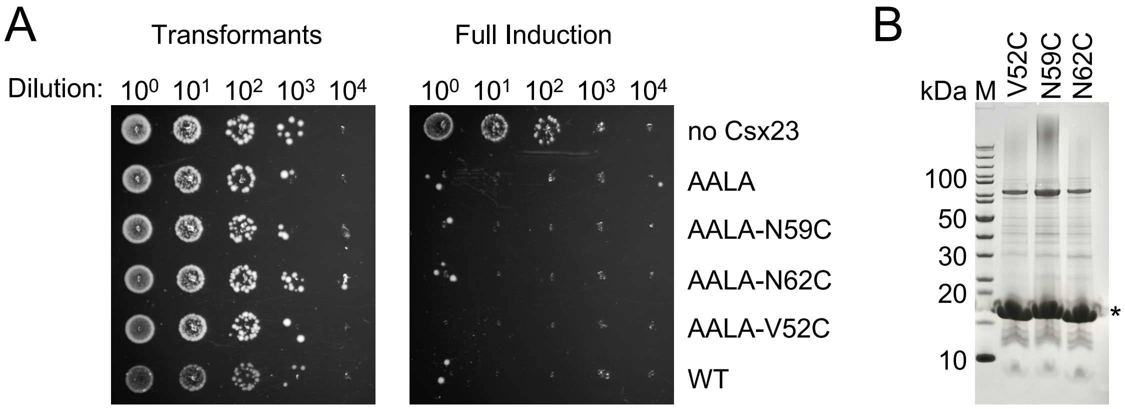

Figure S8. Characterisation of Csx23 variants used for spin-labeling and EPR experiments. **A:** Plasmid challenge assay, demonstrating that all variants retained biological function. Figure representative of two technical replicates. **B:** SDS-PAGE of purified variants of Csx23.

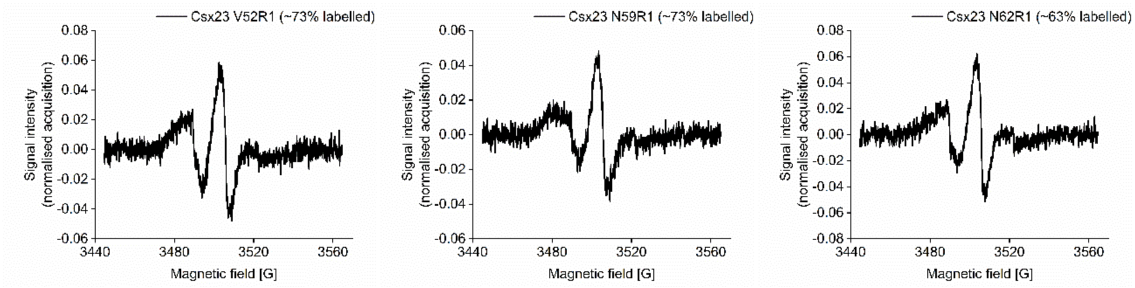

Figure S9. Continuous wave (CW) EPR spectra. Individual CW EPR spectra for MTSL-labelled Csx23 variants as indicated. Details of the constructs and corresponding labelling efficiencies are given on each plot.

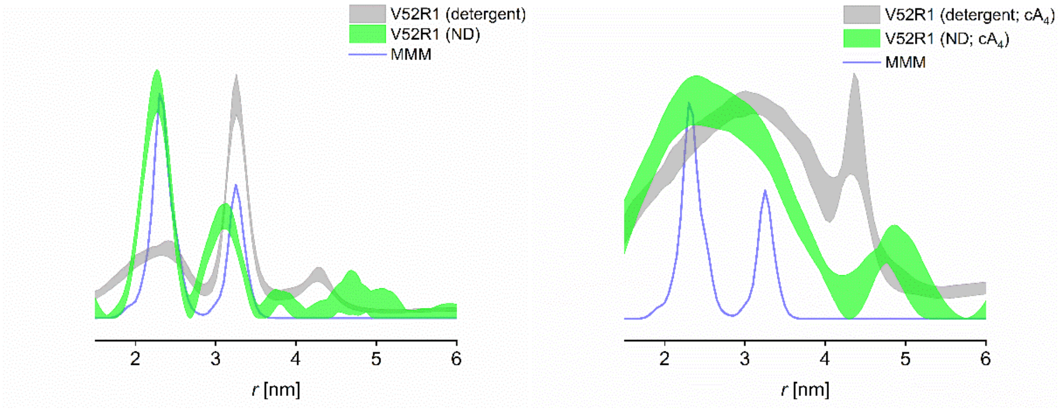

Figure S10. PELDOR data for the Csx23 AALA V52R1 mutant in absence (left) and presence (right) of cyclic nucleotide (cA$_4$), comparing micellar protein (detergent) to protein reconstituted in nanodiscs (ND). The predicted distribution based on the AF2 tetramer predicted structure of Csx23 is shown in blue.

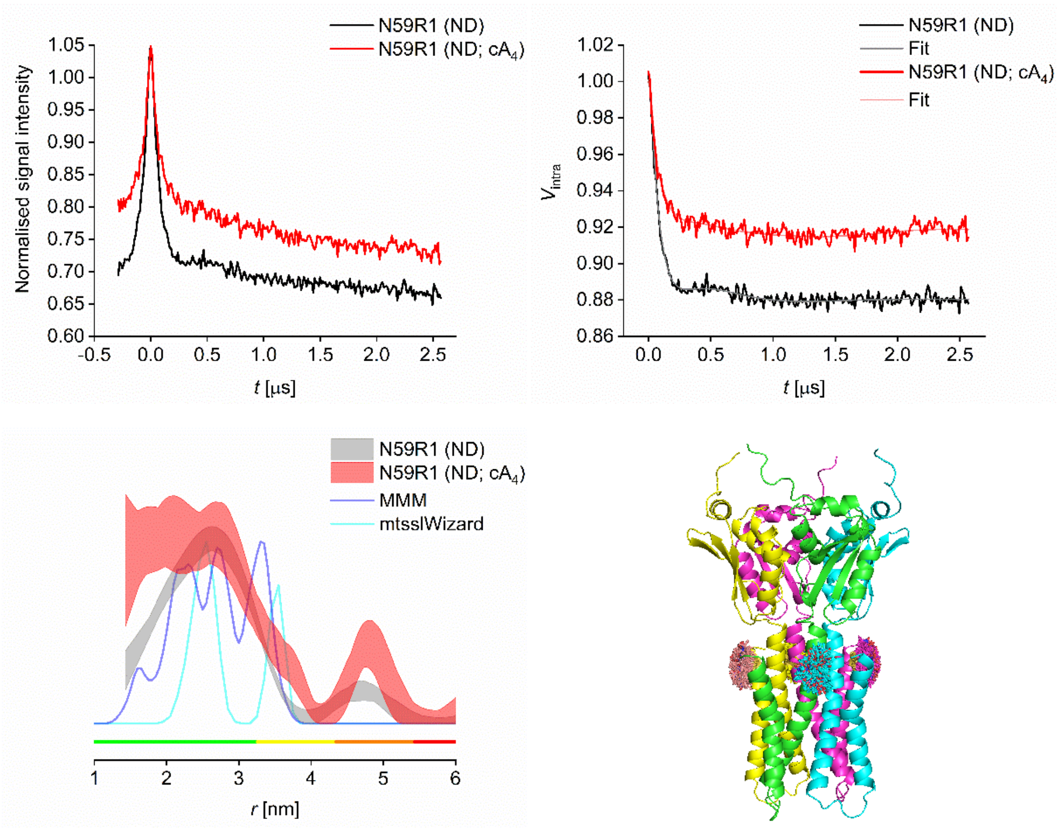

Figure S11. PELDOR data for the Csx23 AALA N59R1 mutant reconstituted in nanodiscs (ND) in presence (red) and absence (grey) of cyclic nucleotide (cA_4). Raw PELDOR data (top left) and background-corrected traces with fits (top right); overlay of corresponding distance distributions shown as 95% confidence bands with predicted distributions from MMM and mtsslWizard based on AF2 predicted structure (bottom left), colour bars indicate reliability ranges (green: shape reliable; yellow: mean and width reliable; orange: mean reliable; red: no quantification possible); cartoon representation of AF2 predicted tetrameric structure of the spin-labelled Csx23 AALA N59R1 tetramer (bottom right).

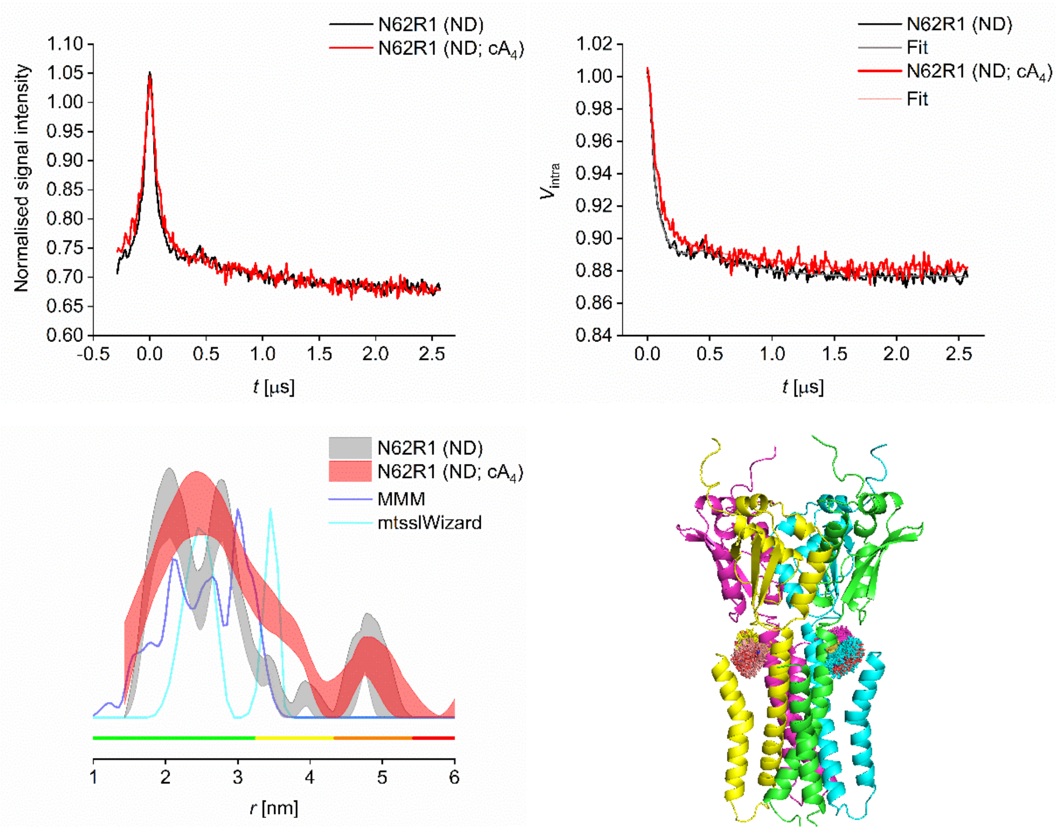

Figure S12. PELDOR data for the Csx23 AALA N62R1 mutant reconstituted in nanodiscs (ND) in presence (red) and absence (grey) of cyclic nucleotide (cA_4). Raw PELDOR data (top left) and background-corrected traces with fits (top right); overlay of corresponding distance distributions shown as 95% confidence bands with predicted distributions from MMM and mtsslWizard based on AF2 structure (bottom left), colour bars indicate reliability ranges (green: shape reliable; yellow: mean and width reliable; orange: mean reliable; red: no quantification possible); cartoon representation of AF2 predicted tetrameric structure of the spin-labelled Csx23 AALA N62R1 tetramer (bottom right).

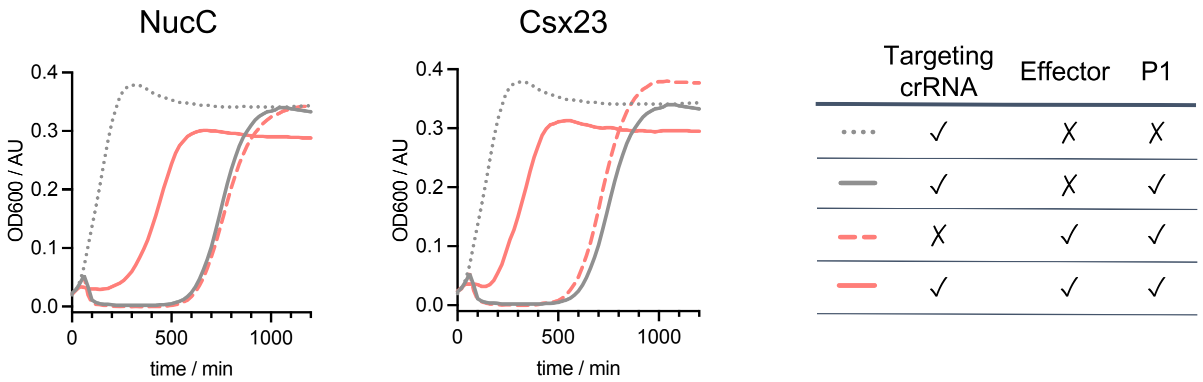

Figure S13. Phage P1 immunity assay using the VmeCmr / effector system. The growth of cells harbouring either Csx23 or NucC in the absence of targeting crRNA (dashed, salmon line) is the same as that of cells not carrying an effector (solid, grey line). The combination of targeting crRNA and effector results (solid, salmon line) in significantly faster recovery of the culture at high MOIs (MOI 15).

Table S1. VmeCRISPR repeat and spacer sequences used in this study.

Description	Sequence (5' to 3')
Vme CRISPR repeat	GTTCACTGCCGCACAGGCAGCTTAGAAA
pUC19 MCS-targeting spacer	GAATTCGAGCTCGGTACCCGGGGATCCTCTAG
phage P1 *lpa*-targeting spacer	TGAGTGATTTATTTCCATGAAGTGGCGTCCCT
pACE2 *tetR*-targeting spacer	GTGCCGAGGATGACGATGAGCGCATTGTTAGA

Table S2. Key to Comparative DeerAnalyzer (CDA2.0) Reports. ND = reconstituted into nanodiscs.

CDA report	Csx23 sample
220913_KAq200.16_DEER_128032_pi_half_ comparative_DEER_analyzer_report	Csx23 AALA V52R1
220913_KAq200.19_DEER_128032_pi_half_ comparative_DEER_analyzer_report	Csx23 AALA V52R1 + cA_4
230210_KAq204.3_PELDOR_128032_pi_half_ comparative_DEER_analyzer_report	Csx23 AALA V52R1, ND
230211_KAq204.6_PELDOR_128032_pi_half_ comparative_DEER_analyzer_report	Csx23 AALA V52R1 + cA_4, ND
230309_KAq210.3_deer_128032_pi_half_ comparative_DEER_analyzer_report	Csx23 AALA N59R1, ND
230312_KAq210.12_DEER_128032_pi_half_ comparative_DEER_analyzer_report	Csx23 AALA N59R1 + cA_4, ND
230310_KAq210.6_DEER_128032_pi_half_ comparative_DEER_analyzer_report	Csx23 AALA N62R1, ND
230311_KAq210.9_DEER_128032_pi_half_ comparative_DEER_analyzer_report	Csx23 AALA N62R1 + cA_4, ND

Table S3. Data collection and refinement statistics for the structure of Csx23 CTD in complex with cA_4.

**Data Collection**	
Space group	*I* 4
Cell dimensions	
a, b, c (Å)	66.4, 66.4, 42.5
α, β, γ (°)	90, 90, 90
Resolution (Å)*	46.97 – 1.76 (1.81 – 1.76)
R_{merge}*	0.08 (1.66)
$I/\sigma(I)$*	14.3 (1.4)
Completeness (%)*	89.1 (56.5)
Multiplicity*	13.3 (14.0)
CC1/2*	1.000 (0.72)
V_m ($Å^3$/Da)	2.13
Solvent (%)	42.4

**Refinement**	
Unique reflections	5251
R_{work} / R_{free}	24.3 / 27.2
RMSD** bonds (Å) / angles (°)	0.032 / 1.110
No. atoms:	
Protein	619
Water	45
cA_4	88
Acetate	6
Sodium	1
B factors ($Å^2$):	
Protein	48.9
Water	45.3
cA_4	24.4
Acetate	34.7
Sodium	28.3
Ramachandran plot: favoured / outlier (%)	96.3 / 0
Molprobity score / centile	1.47 / 95

* Values in parentheses correspond to data in the high resolution shell
** RMSD, root mean square deviation
